# Local knowledge: Who cares?

**DOI:** 10.1186/1746-4269-7-35

**Published:** 2011-11-23

**Authors:** Ina Vandebroek, Victoria Reyes-García, Ulysses P de Albuquerque, Rainer Bussmann, Andrea Pieroni

**Affiliations:** 1 Institute of Economic Botany, The New York Botanical Garden, 2900 Southern Boulevard, 10458 Bronx, New York, USA; 2 ICREA and Institut de Ciència i Tecnologia Ambientals, Universitat Autònoma de Barcelona, 08193 Bellatera, Barcelona, Spain; 3 Universidade Federal Rural de Pernambuco, Departamento de Biologia, Área de Botânica, R. Dom Manoel de Medeiros, S/N, Dois Irmãos, Recife, Pernambuco, 52171-900, Brazil; 4 William L. Brown Center, Missouri Botanical Garden, St. Louis, MO 63110, USA; 5 University of Gastronomic Sciences, Via Amedeo di Savoia 8, I-12060 Pollenzo/Bra, Italy

## Think globally, act locally

Local Knowledge Systems (LKS) consist of the knowledge, beliefs, traditions, practices, institutions, and worldviews developed and sustained by indigenous and local communities, and are believed to represent an adaptive strategy to the environment in which these communities live. The value of LKS has been contested by some scholars as being restricted to local issues [1], and local knowledge holders have alternative been labeled as "guardians of the earth", "conservationists", or as subsistence consumers who will no longer coexist sustainably with the environment when their populations increase, and as they become more integrated into market economies [2-4]. LKS have sometimes been viewed as "traditional", with the negative connotation of being outdated or primitive, and thus of little use to solve problems of modern society [5]. Others have put forward that the adaptive nature, applicability and value of LKS need to be empirically tested and validated by science [6,7].

In this Thematic Series, we focus on the potential and actual value of LKS to local and global challenges. The catchphrase of this paragraph, "Think globally, act locally," has been around for a few decades as a slogan for activism at the local level to increase overall well-being on earth. In an increasingly globalized society with many social, economic and environmental uncertainties, what are the lessons that can be drawn from LKS? One of the virtues of LKS is that these systems depart from the premise of interconnectedness and embeddedness, whereby humans and their behaviors are seen as part of a broader environmental, socio-cultural and spiritual context. The ethnobiological literature showcases several examples that demonstrate the importance and usefulness of LKS for community health, nutrition, education and cultural heritage, conservation and other societal challenges, as we will review in the next paragraphs. This Thematic Series of the *Journal of Ethnobiology and Ethnomedicine *contains solicited manuscripts based on new research that continues to demonstrate the potential and established value of ethnobiological knowledge and its associated plant and animal resources for local communities and society at large, especially in the areas of community health, education and conservation.

## Community Health

The value of LKS for global healthcare has been demonstrated at different levels: (a) in providing primary healthcare to the rural poor in the absence of (mainstream) biomedical healthcare; (b) in being a more satisfying healthcare option to many transnational migrants as compared to biomedical healthcare; and (c) as a way to select interesting candidate plant species for the discovery and development of new pharmaceuticals.

Mainstream (biomedical) healthcare is often lacking in quantity and quality in poor rural areas around the world. Innovative thinking and novel approaches are therefore urgently needed to fill the gap between the need for healthcare and its provision *in situ*. Traditional medicine, including the use of plants, animals and minerals, is a LKS instrumental in providing primary healthcare in many rural areas [8,9]. McDade et al. [10] demonstrated the importance of maternal local plant knowledge for child health in the Bolivian Amazon by showing that mothers who had more plant knowledge also had healthier children. Loss of this type of knowledge is likely to have negative consequences for community health. Collaboration with traditional healers has been suggested as a more rational and cost-effective use of primary healthcare [11,12], and methods and best practices have been put forward to assess the safe and rational use of traditional healers and their plant-based therapeutic practices in the field [13,14]. However, in spite of the recognition of traditional healers as important healthcare actors, obstacles for collaboration with traditional healers are commonly present, including lack of policy support (legislation), and lack of trust in traditional healers by individual biomedical practitioners [11]. Only a handful studies address the conditions for the safe and effective use of traditional healthcare and this topic merits being a priority on research and policy agendas.

Even in places where mainstream (biomedical) healthcare is physically more available, there still is no guarantee that all people will make exclusive use of this system leaving healthcare based on LKS completely behind. For example, in Brazil, the Fulni-ô have developed an intermedical healthcare system that represents a hybridization between local traditional medicine from different cultural groups and biomedicine [15]. Other research has shown the continued use of traditional medicine by residents in main cities (such as in Trujillo, Peru, [16,17]), and even by international migrants in large multicultural centers where biomedical healthcare is readily available. International migrants are often confronted with disparities in healthcare and research has identified several economic, linguistic, and cultural barriers to biomedical healthcare utilization. As a consequence, informal systems of traditional healthcare --including self-treatment with home remedies and consultation with traditional healers-- frequently coexist in parallel with biomedical healthcare. This phenomenon has been observed in New York City, London and Amsterdam [18-20]. New York City, for example, has hundreds of *botánicas*, herbal shops run by Latino immigrants that sell good luck amulets, potions, spiritual candles, saints, and medicinal plants for healing and well-being, and offer consultations with traditional and spiritual healers [21]. The presence and popularity of these *botánicas *underscores their importance for immigrant health and may be demonstrative of their functioning as a coping strategy for psychosocial pressures associated with migration. It is possible that the act of using herbs for healing is complementary to the act of "caring" by traditional healers as a more holistic and satisfying approach to healthcare than biomedicine [21]. Moreover, Moerman and Jonas [22] argue that patients' cultural backgrounds and the relationships they have with their (biomedical or traditional) healthcare providers can directly affect their physiological response to medicines (called the "meaning effect"). A survey among Surinamese immigrants in The Netherlands showed that only a minority of interviewees was positive about the attitude of biomedical staff towards their patients [19]. Studies such as these can help explain the continued popularity of LKS and traditional medicine by migrants and other communities in urbanized settings.

The value of LKS for the historical discovery of plant-derived pharmaceuticals and drug development is extensively discussed in the literature [23-26]. Several challenges have been identified to this approach, such as the lack of new drugs being developed through ethnobotanical research [27], the accelerated loss of medicinal plant species, ecosystems, and their associated LKS, as well as ownership issues related to the use of unimproved genetic and biochemical resources [28]. A major obstacle for community health in developing countries is that drug development in the West has not been generally directed to target diseases that mainly affect developing countries, such as for infectious and parasitic diseases that make up 90% of the disease burden in many of these countries [29].

The paper by Mafimisebi and coworkers in this Thematic Series draws attention to a field related to community health: that of managing the health of livestock through ethnoveterinary practices in Nigeria. These researchers identified variables that were positively associated with the use of traditional medicine for animal health, including farmer's age, household size, and extent of travels, whereas negative associations were found between medicinal plant use, farm income, and the size of livestock. This kind of studies expands previous research with a more narrow focus on human health to a more broad and encompassing framework of health that is inclusive to other parts of community well-being and livelihood strategies, such as ethnoveterinary healthcare.

## Nutrition

LKS hold potential value to identify plants for diversification of human dietary habits. Modern diets are generally poor in plant diversity, and diet-related diseases have become a large burden on global economies. The diversity of food crops is very low in comparison with the global diversity of edible plants. There exist an estimated 50,000 edible plant species. In comparison, there are an estimated 250-300 food crops, with the number of plant species calculated to "feed the world" as ranging between 7 and 103 plants [30]. The ethnobotanical literature is rich with evidence of the wide diversity of wild, weedy, and semi-domesticated plant foods as well as non-commercial landraces eaten around the globe. These plant species often grow in environments influenced by human activity, including agricultural areas and ruderal places (waste grounds) and have multiple uses, including food, medicine, and forage [31-33]. Examples include *liakra *in Italy, *quelites *in Mexico, *tiusinte *(*Dioon mejiae *Standl. & L.O. Williams, Zamiaceae) in Honduras, bush foods in Australia, landraces in the Catalonian Pyrenees, as well as famine foods, vegetable snacks and children's foods in many countries, [33-40]. These plants are important traditional components of rural diets worldwide, even though their use is sometimes stigmatized as symbolic of poverty [32]. They present opportunities for direct nutritional supplementation as part of a balanced and varied diet, contribute to health maintenance as functional or medicinal foods, and sometimes supply a modest monetary income [32,34,41]. The so called "Mediterranean diet", for example, includes consumption of an impressive number of wild and weedy vegetables in many rural areas [42,43], which are often overlooked by nutritional studies.

Previous research among adults from an indigenous Amazonian society in Bolivia suggests that LKS are associated with improvement of nutritional status, measured through the Body Mass Index (BMI) [44]. This association was stronger for unschooled adults and for those who lived far from a market town. A plausible explanation for the association between local knowledge and BMI relates to the effectiveness of medicinal plants and the nutritious value of wild, edible plants. Persons with higher knowledge of medicinal and edible plants may have a higher BMI because they can use their knowledge to cure themselves and to safeguard their consumption in times of environmental and/or nutritional shocks. This finding has important implications for the nutritional status of rural and indigenous populations.

## Education and cultural heritage

Given the global decline in languages and cultures worldwide, LKS can be a useful instrument in cultural awareness programs and formal intercultural education curricula to strengthen cultural identity and promote biocultural heritage [38]. In addition, incorporation of ethnobiological knowledge, beliefs and practices in school curricula of rural communities can put scientific learning about the environment within its traditional context, contribute to local well-being and be directly applicable to the life of rural students [45]. Tools such as analysis of folksongs and storytelling have been put forward as less formal and more engaging teaching methods in dissemination of LKS [46,47].

At the beginning of the 1990s, Bolivia was the first country in Latin America to make intercultural education an official state policy. "The rationale behind this intercultural and bilingual education strategy was to provide indigenous children with the self-esteem to achieve better results, while maintaining their communities' cultural values and practices." [48]. This way, new generations could strengthen their cultural identity and be better prepared for intercultural encounters. However, apart from the language issue, the importance of developing and teaching a non-mainstream curriculum that is as relevant as possible to the local cultural context is illustrated by the following example.

While visiting an elementary school in a Trinitario indigenous community in the Bolivian Amazon rainforest in the *Territorio Indígena Parque Nacional Isiboro-Sécure *(TIPNIS) in August 2009, the first author (IV) sat in on a multilingual class taught at the elementary school in the community that covered six Bolivian languages (Spanish, Quechua, Aymara, Yurakaré, Trinitario, Tsimane'). The teacher was of Quechua descent (one of the main highland groups in Bolivia together with the Aymaras) and the class consisted of young Trinitario, and to a lesser extent Yurakaré and Tsimane' students. The teacher followed a book that would have been useful in a Quechua or Aymara context, reciting the Spanish and Quechua/Aymara names for animals, such as "llama" (llama) and "oveja" (sheep), and professions such as "abogado" (lawyer). He then asked the students to mention the equivalent names for these items in Trinitario, Yurakaré, and Tsimane' language. His efforts were not very successful because these animals and professions are not a part of daily life in the lowlands of Bolivia where Yurakarés, Trinitarios and Tsimane' live. Also, the teaching setting (classroom) and teaching method (inquiry) did not seem very conducive to participation from the students. Reyes-García and coworkers [49] have already argued before that a major challenge to incorporating LKS in formal school curricula lies in using culturally representative ways of teaching about local knowledge, for example through field trips, observation and informal instruction.

A few days later, during a one-hour trip to a nearby community for the purchase of goods, some of the young indigenous students displayed an entirely different behavior as compared to the classroom setting. Observing that the author was interested in plants, they spontaneously started showing the plants they knew and pointing to marks left behind by forest animals. Given that these communities are surrounded by secondary and primary forest with an abundance of teaching props, it would not be unthinkable for the teacher to design hands-on classes while taking students for walks around the school. However, the fact that many teachers in these communities belong to a different cultural group than their students, as well as the teachers' training in the formal education system may represent barriers to such initiatives. The Bolivian government seems to recognize that changes need to be made and has initiated a process of regionalization of teaching curricula to make them better adapted to the local context (Alan Lispuerger, pers. comm.). In spite of this worthy initiative, it would be indicated if more indigenous lowland people were to participate in teacher training and become responsible for developing curricula in their own communities.

Formal schooling and knowledge acquired at school have been considered both a cause of loss of indigenous and LKS (because it opens pathways to the non-indigenous world and worldviews) and a potential remedy to its demise (if educational curricula are aligned with indigenous realities through teaching in local languages and incorporation of local knowledge in curricular materials). Although other researchers have found a negative association between formal schooling and local knowledge [50], Reyes-García and coworkers [49] found that among the Tsimane' (a society of forager-horticulturalists in the Bolivian Amazon) contextualized learning actually helped maintain LKS. Research among Tsimane' who had been exposed to a partially contextualized school curriculum for several decades due to the influence of missionaries found that although schooling and academic knowledge had a negative association with local knowledge, this magnitude was low, probably because schooling was partially contextualized. Contrary to many other indigenous children in Bolivia, the Tsimane' educational system was partially contextualized with children taught in Tsimane' language, by Tsimane' teachers using educational materials in Tsimane'. This partially contextualized teaching did not necessarily strengthen LKS, but might have helped to lower the negative effect of schooling on local environmental knowledge. This research offers the policy recommendation that educational programs for local and indigenous communities should place more emphasis on the inclusion of LKS in formal school curricula through contextualized learning [49].

The paper by McCarter and Gavin in this Thematic Series adds to the body of literature on challenges for inclusion of LKS in formal education. In Vanuatu, an island nation located in the South Pacific Ocean, these challenges include: (a) lack of government support; (b) high cultural diversity (when there are many different cultural and language groups); (c) problems associated with ownership over LKS; and (d) difficulties to adapt LKS to formal, academic ways of teaching.

Another opportunity for applying LKS to education lies in the promotion of knowledge of cultural diversity in medical education and the clinical practice. Culture is known to influence the experience and expression of illness, as well as the type of treatment that patients pursue for illnesses, including self-treatment with herbal remedies and use of traditional healers. The study of LKS can shed light on the perceived symptoms, causes and preferred treatments for illnesses that have a strong cultural context, also named folk illnesses or culture-bound syndromes [51]. Ethnobiological research is well placed to inform and train healthcare providers in becoming more culturally sensitive to the knowledge, beliefs and practices of patients from diverse cultures. The use of ethnobiological information in the clinical encounter is pivotal in establishing a relationship of trust between physicians and their minority and often underserved patients, and to promote an environment where the use of herbal remedies by patients is more readily disclosed. Non-disclosure may be potentially harmful, as unforeseen drug-herb interactions are possible if herbal remedies are taken concomitantly with prescribed medications. Unfortunately, many cultural competency training programs in medical schools mostly exists as elective classes and not as full-fledged required teaching programs. Advocacy from medical educators is urgently needed to promote the incorporation of required cultural competency modules into the medical curriculum and in continuing education programs directed at physicians, nurses and other health care professionals.

## Conservation

Human harvesting pressure has been shown to negatively affect the size of the Himalayan snow lotus (*Saussurea laniceps *Hand.-Mazz., Asteraceae), a valuable plant species in Tibetan and Chinese medicine, on a short-term evolutionary scale [52]. On the other hand, it is well-known that moderate human disturbance can enhance biodiversity [2]. LKS often contain elements that are relevant to conservation initiatives, such as the existence of sacred groves [53], social taboos [54] or community embargos [37] that regulate access to and use of biological resources, or the presence of behavioral practices that are conducive to maintaining a high level of biodiversity (such as, for example, in traditionally managed coffee agroecosystems, [55]). In the Catalonian Pyrenees, farmers maintain *in situ *agrobiodiversity in their home gardens through human networks of seed exchange, whereby the position of an individual farmer in the network was positively associated with landrace conservation [56]. Also, women, people over 65 years of age, experienced gardeners, and people who grew their home garden organically were more likely to conserve landraces than people without those characteristics [39].

Community-based conservation is based upon the premise that local development and biodiversity conservation are not necessarily mutually exclusive. Even though the subsistence activities of small-scale farmers and forest dwelling communities may not fit a narrow view of conservation, these cultural groups can be the best natural allies for conservationists [57]. For example, in Brazil, Albuquerque and coworkers [58] carried out a rapid ethnobotanical appraisal to identify culturally important plants for the Fulni-ô people that were highly vulnerable and in need of conservation attention. The authors recommend the direct involvement of the Fulni-ô people in the design and implementation of a management plan for conservation and monitoring of these culturally important vulnerable plant species. Because of the interdisciplinary nature of ethnobiology, scholars in this field are exceptionally well placed to further research on socially-defined conservation and to help developing a pluralistic cross-cultural philosophy of conservation in which local and Western scientific conservation ethics are both taken into account.

Two papers in this Thematic Series are relevant to conservation. Carvalho and Frazão-Moreira advocate that in order for contemporary strategies aimed at preservation of cultural and ecological diversity to be successful, it is necessary to go beyond validating and integrating local people's knowledge and expertise into the design, management and maintenance of protected areas. These authors argue that instead it is necessary to make local people active participants in conservation programs. Newmaster and coworkers examine the depth and breadth of LKS in relation to a threatened ecosystem of seagrasses that is crucial for sustaining the livelihood of local communities. Their research shows that these communities have a much more fine-tuned knowledge of seagrass taxa as compared to the scientific classification system since they recognize five times more taxa (with 10 species representing a total of 50 ethnotaxa), each with their unique roles in the local ecosystem and usefulness to humans. This wealth of cultural information deserves recognition and should be taken into account when developing an action plan for seagrass conservation.

## Conclusions

These days, the popular media frequently report on emerging socio-environmental and health challenges, including climate change, souring food prices due to global food shortages, emergence of new diseases, and accelerated development that threatens the survival of tropical forests. The intrinsic nature of LKS has always been to cope with environmental unpredictability and to find ways to mitigate the effects of this unpredictability through ongoing experimentation with natural resources. Tibetan villagers have detailed perspectives on climate change and its impacts [59]. Jamaican LKS are recognized as the driving force that keeps the domestic food production sector in Jamaica alive [5]. Herbal practitioners in Tanzania have extensive knowledge about the use of traditional medicines to manage opportunistic infections associated with HIV/AIDS [60].

Hence, there is a lot to be learned from LKS, not only from the knowledge itself, but also from the philosophical meanings embedded in that knowledge. The view that humans and nature are not seen as separate from each other, but as mutually dependent, as well as the concept of "mindfulness", are ideologies that would greatly benefit our contemporary approach to life. On the other hand, the use of LKS should not be seen as a panacea to every problem of humanity either, as this might raise expectations about their usefulness to unrealistically high levels, placing high burdens upon the backs of those communities who are often already struggling to survive and maintain their cultural independence. Rather, perhaps LKS should be considered complementary to scientific research. Equal partnership and integration of scientific and local knowledge systems may represent a significant step forward in achieving better applied results. In a recent comment in *Nature*, Huntington [61] argues for establishing better collaborations between scientists and local communities through developing new ways of gathering data pertaining to LKS (for example, by providing local populations with Global Positioning System units so they can learn to record natural phenomena observed in their daily lives) and developing better ways of managing information about LKS (by means of videos, maps and songs instead of spreadsheets). For example, Sami reindeer herders are working together with scientists to document snow conditions and their impact on herding practices under future climate conditions. Huntington also calls upon natural scientists to be more accepting and inclusive of LKS when debating ecological phenomena such as climate change.

All too often, ethnobiology scholars still tend to focus mainly on utilitarian aspects of ethnobiology by publishing inventories of locally used plants, animals and minerals. While this certainly has its own merits in preserving oral local and traditional knowledge (but see also the issue of intellectual property rights raised by [28]), contemporary ethnobiologists can and should go further. Since knowledge is power, the use of LKS can be a mechanism for local empowerment and equal co-partnership in community-based projects. There are no reasons why not more ethnobiologists should adopt this bottom up approach and design their research projects around local needs and challenges in close consultation with local communities. Or, to put it in the words of a *cacique mayor *(local chief) from an Amazon community in a conversation with the first author: "why is it that scientists are coming to the rainforest to study ants and plants, while we are here with nobody worrying about us?"

## References

[B1] Briggs J The use of indigenous knowledge in development: problems and challenges Progress in Development Studies 2005 5 99 114 10.1191/1464993405ps105oa

[B2] Smith EA Wishnie M Conservation and subsistence in small-scale societies Annual Reviews in Anthropology 2000 29 493 524 10.1146/annurev.anthro.29.1.493

[B3] Godoy R Reyes-García V Byron E Leonard WR Vadez V The effect of market economies on the well-being of indigenous peoples and on their use of natural resources Annual Review of Anthropology 2005 34 121 138 10.1146/annurev.anthro.34.081804.120412

[B4] Lu F Integration into the Market among Indigenous Peoples: A Cross-Cultural Perspective from the Ecuadorian Amazon Current Anthropology 2007 48 593 602 10.1086/519806

[B5] Beckford C Barker D The role and value of local knowledge in Jamaican agriculture: adaptation and change in small-scale farming The Geographical Journal 2007 173 118 128 10.1111/j.1475-4959.2007.00238.x

[B6] Alexiades MN Ethnobotany in the third millennium: Expectations and unresolved issues Delpinoa 2003 45 15 28

[B7] Albuquerque UP Hanazaki N Five problems in current ethnobotanical research--and some suggestions for strengthening them Human Ecology 2009 37 653 661 10.1007/s10745-009-9259-9

[B8] Vandebroek I Calewaert J-B De Jonckheere S Sanca S Semo L Van Damme P Van Puyvelde L De Kimpe N Use of medicinal plants and pharmaceuticals by indigenous communities in the Bolivian Andes and Amazon Bulletin of the World Health Organization 2004 82 243 250 15259252 PMC2585970

[B9] Alves RRN Rosa IML Biodiversity, traditional medicine and public health: where do they meet? Journal of Ethnobiology and Ethnomedicine 2007 3 14 10.1186/1746-4269-3-14 17376227 PMC1847427

[B10] McDade TW Reyes-García V Blackinton P Tanner S Huanca T Leonard WR Ethnobotanical knowledge is associated with indices of child health in the Bolivian Amazon Proceedings of the National Academy of Sciences of the United States of America 2007 104 6134 6139 10.1073/pnas.0609123104 17389376 PMC1851091

[B11] Kaboru BB Falkenberg T Ndubani P Höjer B Vongo R Brugha R Faxelid E Can biomedical and traditional health care providers work together? Zambian practitioners' experiences and attitudes towards collaboration in relation to STIs and HIV/AIDS care: a cross-sectional study Human Resources for Health 2006 4 16 10.1186/1478-4491-4-16 16846497 PMC1540435

[B12] Mignone J Bartlett J O'Neil J Orchard T Best practices in intercultural health: five case studies in Latin America Journal of Ethnobiology and Ethnomedicine 2007 3 31 10.1186/1746-4269-3-31 17803820 PMC2000867

[B13] Graz B Assessing traditional healers: an observational clinical study of classical Arabic medicine in Mauritania, with comparison of prognosis and outcome Tropical Doctor 2005 35 217 218 10.1258/004947505774938684 16354473

[B14] Graz B Elisabetsky E Falquet J Beyond the myth of expensive clinical study: Assessment of traditional medicines Journal of Ethnopharmacology 2007 113 382 386 10.1016/j.jep.2007.07.012 17728084

[B15] Soldati GT Albuquerque UP Ethnobotany in intermedical spaces: The case of the Fulni-ô Indians (Northeastern Brazil) Evidence-Based Complementary and Alternative Medicine 2012 10.1155/2012/648469 PMC3180078 21961025

[B16] Bussmann RW Sharon D Lopez A Blending traditional and Western medicine: Medicinal plant use amongst patients at Clinica Anticona in El Porvenir, Peru Ethnobotany Research and Applications 2007 5 185 199

[B17] Bussmann RW Sharon D Garcia M From chamomile to aspirin? Medicinal plant use among clients at Laboratorios Beal in Trujillo, Peru Ethnobotany Research and Applications 2009 7 399 407

[B18] Ceuterick M Vandebroek I Pieroni A Resilience of Andean urban ethnobotanies. A comparison of medicinal plant use among Bolivian and Peruvian migrants in the United Kingdom and in their countries of origin Journal of Ethnopharmacology 2011 136 27 54 10.1016/j.jep.2011.03.038 21470576

[B19] Van Andel T Westers P Why Surinamese migrants in the Netherlands continue to use medicinal herbs from their home country Journal of Ethnopharmacology 2010 127 694 701 10.1016/j.jep.2009.11.033 20004237

[B20] Vandebroek I Balick MJ Ososki A Kronenberg F Yukes J Wade C Jiménez F Peguero B Castillo D The importance of botellas and other plant mixtures in Dominican traditional medicine Journal of Ethnopharmacology 2010 128 20 41 10.1016/j.jep.2009.12.013 20006697 PMC2829983

[B21] Viladrich A Botánicas in America's backyard: Uncovering the world of Latino healers' herb-healing practices in New York City Human Organization 2006 65 407 419

[B22] Moerman DE Jonas WB Deconstructing the placebo effect and finding the meaning response Annals of Internal Medicine 2002 136 471 476 11900500 10.7326/0003-4819-136-6-200203190-00011

[B23] Farnsworth NR Akerele O Bingel AS Soejarto DD Guo Z Medicinal plants in therapy Bulletin of the World Health Organization 1985 63 965 981 3879679 PMC2536466

[B24] Araújo TAS Alencar NL Amorim ELC Albuquerque UP A new approach to study medicinal plants with tannins and flavonoids contents from the local knowledge Journal of Ethnopharmacology 2008 120 72 80 10.1016/j.jep.2008.07.032 18725282

[B25] McClatchey WC Mahady GB Bennett BC Shiels L Savo V Ethnobotany as a pharmacological research tool and recent developments in CNS-active natural products from ethnobotanical sources Pharmacology & Therapeutics 2009 123 239 254 19422851 10.1016/j.pharmthera.2009.04.002 PMC2700180

[B26] Queiroz Siqueira CF Vasconcelos Cabral DL da Silva Peixoto Sobrinho TJ Cavalcanti de Amorim EL Gomes de Melo J Sousa Araújo TA Albuquerque UP Levels of tannins and flavonoids in medicinal plants: Evaluating bioprospecting strategies Evidence-Based Complementary and Alternative Medicine 2012 10.1155/2012/434782 PMC3182569 21969842

[B27] McClatchey W Medicinal bioprospecting and ethnobotany research Ethnobotany Research & Applications 2005 3 189 19022115750

[B28] Shingu GK Ownership and sustainability issues of botanical medicines Ethnobotany Research & Applications 2005 3 17 2322115750

[B29] Nyigo VA Malebo HM Drug discovery and developments in developing countries: bottlenecks and way forward Tanzania Health Research Bulletin 2005 7 154 158 16941941 10.4314/thrb.v7i3.14253

[B30] Prescott-Allen R Prescott-Allen C How many plants feed the world? Conservation Biology 1990 4 365 374 10.1111/j.1523-1739.1990.tb00310.x

[B31] Stepp JR Moerman D The importance of weeds in ethnopharmacology Journal of Ethnopharmacology 2001 75 19 23 10.1016/S0378-8741(00)00385-8 11282438

[B32] Price LL Pieroni A, Price L Wild food plants in farming environments with special reference to Northeast Thailand, food as functional and medicinal, and the social roles of women Eating and Healing: Traditional food as medicine 2006 Binghamton, New York: The Haworth Press 65 99

[B33] Vandebroek I Sanca S Pieroni A, Price L Food medicines in the Bolivian Andes (Apillapampa, Cochabamba Department) 2006 Eating and Healing: Traditional food as medicine 2006 Binghamton, New York: The Haworth Press 273 295

[B34] Vieyra-Odilon L Vibrans H Weeds as crops: The value of maize field weeds in the valley of Toluca, Mexico Economic Botany 2001 55 426 443 10.1007/BF02866564

[B35] Pieroni A Nebel S Quave C Munz H Heinrich M Ethnopharmacology of liakra: traditional weedy vegetables of the Arbereshe of the Vulture area in southern Italy Journal of Ethnopharmacology 2002 81 165 185 10.1016/S0378-8741(02)00052-1 12065148

[B36] Pardo-de-Santayana M Tardío J Morales R The gathering and consumption of wild edible plants in the Campoo (Cantabria, Spain) International Journal of Food Sciences and Nutrition 2005 56 529 542 10.1080/09637480500490731 16503563

[B37] Bonta M Flores Pinot O Graham D Haynes J Sandoval G Ethnobotany and conservation of tiusinte (*Dioon mejiae *Standl. & L.O. Williams, Zamiaceae) in Northeastern Honduras Journal of Ethnobiology 2006 26 228 257 10.2993/0278-0771(2006)26[228:EACOTD]2.0.CO;2

[B38] Cruz García GS The mother-child nexus. Knowledge and valuation of wild food plants in Wayanad, Western Ghats, India Journal of Ethnobiology and Ethnomedicine 2006 2 39 10.1186/1746-4269-2-39 16968535 PMC1613234

[B39] Calvet-Mir L Calvet-Mir M Vaqué-Nuñez L Reyes-García V Landraces in situ conservation: A case study in high-mountain home gardens in Vall Fosca, Catalan Pyrenees, Iberian Peninsula Economic Botany 2011 65 146 157 10.1007/s12231-011-9156-1

[B40] Menendez-Baceta G Aceituno-Mata L Tardío J Reyes-García V Pardo-de-Santayana M Wild edible plants traditionally gathered in Gorbeialdea (Biscay, Basque Country) Genetic Resources and Crop Evolution in press

[B41] Nascimento VT Moura NP Silva Vasconcelos MA Sucupira Maciel MI Albuquerque UP Chemical characterization of native wild plants of dry seasonal forests of the semi-arid region of northeastern Brazil Food Research International 2011 44 2112 2119 10.1016/j.foodres.2010.12.024

[B42] Pieroni A Nebel S Santoro RF Heinrich M Food for two seasons: culinary uses of non-cultivated local vegetables and mushrooms in a south Italian village International Journal of Food Sciences and Nutrition 2005 56 245 272 10.1080/09637480500146564 16096136

[B43] Hadjichambis A Paraskeva-Hadjichambi D Della A Giusti ME De Pasquale C Lenzarini C Censorii E Gonzales-Tejero M Sanchez-Roja C Ramiro-Gutierrez J Skoula M Johnson C Sarpaki A Hmamouchi M El-Johri S El-Demerdash M El-Zayat M Pieroni A Wild and semi-domesticated food plants consumption in seven circum-Mediterranean areas International Journal of Food Sciences and Nutrition 2008 59 383 414 10.1080/09637480701566495 18979618

[B44] Reyes-García V McDade T Vadez V Huanca T Leonard W Tanner S Godoy R Non-market returns to traditional human capital: Nutritional status and traditional knowledge in a native Amazonian society Journal of Development Studies 2008 44 206 221

[B45] Kraipeerapun K Thongthew S The development of ethnobotany curriculum for students in rural schools: An approach that incorporates the needs and insights of local communities International Education Journal 2007 8 64 70

[B46] Armstrong M Kimmerer RW Vergun J Education and research opportunities for traditional ecological knowledge Frontiers in Ecology and the Environment 2007 5 W12 W14 10.1890/1540-9295(2007)5[w12:EAROFT]2.0.CO;2

[B47] Mekbib F Folksong based appraisal of bioecocultural heritage of sorghum (*Sorghum bicolor *(L.) Moench): A new approach in ethnobiology Journal of Ethnobiology and Ethnomedicine 2009 5 19 10.1186/1746-4269-5-19 19575802 PMC2717052

[B48] Lopes Cardozo MTA Future teachers and social change in Bolivia. Between decolonization and demonstration 2011 Delft, The Netherlands: Uitgeverij Eburon

[B49] Reyes-García V Kightley E Ruiz-Mallén I Fuentes-Pelaez N Demps K Huanca T Martínez-Rodríguez MR Schooling and local ecological knowledge: Do they complement or substitute each other? International Journal of Educational Development 2010 30 305 313 10.1016/j.ijedudev.2009.11.007

[B50] Sternberg R Nokes C Geissler P Prince R Okatcha F Bundy D Grigorenko E The relationship between academic and practical intelligence: a case study in Kenya Intelligence 2001 29 401 418 10.1016/S0160-2896(01)00065-4

[B51] Vandebroek I Thomas E Sanca S Van Damme P Van Puyvelde L De Kimpe N Comparison of health conditions treated with traditional and biomedical healthcare in a Quechua community in rural Bolivia Journal of Ethnobiology and Ethnomedicine 2008 4 1 10.1186/1746-4269-4-1 18194568 PMC2265262

[B52] Law W Salick J Human-induced dwarfing of Himalayan snow lotus, *Saussurea laniceps *(Asteraceae) Proceedings of the National Academy of Sciences of the United States of America 2005 102 10218 10220 10.1073/pnas.0502931102 16006524 PMC1177378

[B53] Lebbie AR Guries RP Ethnobotanical Value and Conservation of Sacred Groves of the Kpaa Mende in Sierra Leone Economic Botany 1995 49 297 308 10.1007/BF02862349

[B54] Colding J Folke C "Invisible" systems of local resource management and biological conservation Ecological Applications 2001 11 584 600

[B55] Moguel P Toledo VM Biodiversity conservation in traditional coffee systems of Mexico Conservation Biology 1999 13 11 21 10.1046/j.1523-1739.1999.97153.x

[B56] Calvet-Mir L Calvet-Mir M Molina JL Reyes-García V Seeds exchange as an agrobiodiversity conservation mechanism: A case study in Vall Fosca, Catalan Pyrenees, Iberian Peninsula Ecology and Society in press

[B57] Berkes F Rethinking community-based conservation Conservation Biology 2004 18 621 630 10.1111/j.1523-1739.2004.00077.x

[B58] Albuquerque UP Soldati GT Sieber SS Medeiros PM Sá JC Souza LC Rapid ethnobotanical diagnosis of the Fulni-ô Indigenous lands (NE Brazil): floristic survey and local conservation priorities for medicinal plants Environment, Development and Sustainability 2011 13 277 292 10.1007/s10668-010-9261-9

[B59] Byg A Salick J Local perspectives on a global phenomenon--Climate change in Eastern Tibetan villages Global Environmental Change 2009 19 156 166 10.1016/j.gloenvcha.2009.01.010

[B60] Kisangau DP Lyaruu HVM Hosea KM Joseph CC Use of traditional medicines in the management of HIV/AIDS opportunistic infections in Tanzania: A case in the Bukoba rural district Journal of Ethnobiology and Ethnomedicine 2007 3 29 10.1186/1746-4269-3-29 17623081 PMC1941724

[B61] Huntington HP The local perspective Nature 2011 478 182 183 10.1038/478182a 21993743

